# Effect of breast milk intake volume on early behavioral neurodevelopment of extremely preterm infants

**DOI:** 10.1186/s13006-024-00612-5

**Published:** 2024-01-17

**Authors:** Ying Gao, Xiaoyu Lu, Mengqing Pan, Chuntian Liu, Yuxiao Min, Xiaochun Chen

**Affiliations:** The Second Affiliated Hospital and Yuying Children’s Hospital of Wenzhou Medical University, Wenzhou, China

**Keywords:** Extremely premature infant, Neonate, Neurodevelopment, Breastfeeding, Nneonatal care, Intracranial hemorrhage

## Abstract

**Background:**

This study aimed to explore the effects of breast milk feeding volume on the early behavioral neurodevelopment of extremely preterm infants (gestational age < 28 weeks).

**Methods:**

The study was conducted from 1 January 2021 to 31 March 2023. A total of 187 preterm infants from a neonatal intensive care unit (NICU) in a Grade III Class A hospital in Zhejiang, China, were divided based on the proportion of breast milk in their total enteral nutrition: high proportion (≥ 80%, including exclusive breast milk feeding), medium proportion (20% ~ < 80%), and low proportion (< 20%). The study investigated motor performance and behavioral neurodevelopment at 37 weeks of corrected gestational age, as well as the total incidence of intracranial hemorrhage within the first four weeks postpartum.

**Results:**

The low breast milk feeding group had significantly lower scores in infant motor performance (31.34 ± 5.85) and elicited item scores (19.89 ± 5.55) compared to the medium and high groups (33.52 ± 4.33, 22.13 ± 4.22; and 35.86 ± 5.27, 23.91 ± 4.98), *p* < 0.05, respectively. Despite no significant difference in behavioral ability, the low proportion group exhibited lower passive muscle tension and primitive reflex scores than the medium and high proportion groups. The high proportion group showed higher active muscle tension scores. Ultrasound results revealed varying incidences of intracranial hemorrhage: 72.9% in low, 52.5% in medium, and 19.6% in the high proportion groups.

**Conclusions:**

Medium to high levels of breast milk feeding contribute positively to motor and behavioral neurological development in extremely preterm infants and decrease the likelihood of ventricular hemorrhage. However, it does not have a significant effect on the development of behavioral abilities. Due to the limited sample size, the next step will be to expand the sample size and further investigate the extent of the impact on various aspects of the nervous system.

## Background

The World Health Organization defines newborns with a gestational age of less than 37 weeks as premature infants and those younger than 28 weeks are called extreme preterm infants (EPIs) [1]. There are 15 million premature babies born in the world every year, 7.8% of which occur in China [2], and EPIs constitute 5.2% of the total global premature baby population [3]. Due to the small gestational age of EPIs at birth, the development of all systems is extremely immature, and most of the infants need to be admitted to the neonatal intensive care unit (NICU) for treatment after birth. Also, they are disturbed by external environmental stimuli and stimulated by various invasive operations [4], which can easily cause growth and developmental lag, especially to neurological development [5]. A meta-analysis showed that 52% of surviving EPIs worldwide showed some degree of neurodevelopmental impairment [6]. Studies have shown that the breast milk of preterm mothers contains micronutrients, hormones, immune components and other metabolites that not only promote the continuous maturation and development of the systemic immune system, but also improve and promote the rapid maturation of the behavioral nervous system of preterm infants [7]. The effects on the nervous system can extend from infancy to school age and even adolescence, and protect the neurodevelopment and cognitive function of preterm infants [8]. However, the current rate of exclusive breast milk feeding for hospitalized preterm infants in China is less than 19% [9]. Therefore, the aim of this study was to investigate the effect of different breast milk volumes on the behavioral neurodevelopment of EPIs.

## Methods

### Study design

A retrospective study was conducted on EPIs born in the obstetrics department of a tertiary hospital in Wenzhou from 1 January 2021 to 31 March 2023 and admitted to NICU. Inclusion criteria were: ① Gestational age < 28 weeks; ② Apgar scores at one minute, five minutes and ten minutes after birth were 8–10 points(muscle tone not deducted [10]); ③ Transferred directly to the NICU after birth; ④ Early enteral nutrition (EN) and education for family members on breast milk feeding within 12 h after birth [11];⑤ Oxygen saturation is maintained above 88%. Exclusion criteria comprised: ① Mothers with contraindications to breastfeeding; ② Infant born with complications such as (congenital malformations or genetic metabolic diseases; cystic periventricular leukomalacia); ③ Children who were re-admitted to hospital (those who were readmitted within one month after being discharged). Further exclusion criteria were: ① Infant with fasting due to illness; ② treatment interruption, or transfer to another hospital for various reasons.

This study was approved by the Ethics Committee of the Second Affiliated Hospital of Wenzhou Medical University· Yuying Children ‘s Hospital (2023-K-29-01).

### Calculation of sample size

According to the retrospective design, this study adopts G.Power to calculate the sample size, the effect size is set to 0.3, the test efficiency is set to 0.9, and *α* is set to 0.05. The total sample size of the three groups is calculated as *n* = 144 cases, allowing for a 20% drop out rate for the sample. In the end, at least three groups of research subjects with a total of 172 cases will be needed.

### Breast milk feeding

According to the early EN of premature infants and the classification of breast milk feeding [11], breast milk feeding was divided into three groups: high proportion, medium proportion and low proportion of breast milk feeding. Within the groups, the proportion of breast milk feeding comprising the total feeding amount was ≥ 80% for the high proportion of breast milk feeding (including exclusive breast milk feeding) group; 20% ~ < 80% of breast milk feeding for the medium proportion group; and, < 20% for the low proportion of breast milk feeding group. In the NICU, the breast milk volume for each EPIs is documented using the electronic medical record system. The proportion of breast milk volume is defined as the percentage of breast milk intake in the total EN consumed up to 37 weeks of corrected age.

### Nutrition plan

Breast milk is provided immediately once available. When the mother delivers breast milk to the NICU, it is the preferred source of infant nutrition. Infants with inadequate breast milk intake are supplemented with preterm infant formula, with a preference for breastfeeding if breast milk is available. When the total breast milk feeding volume reaches 50–80 mL / kg / d, breast milk fortifier is added. Breast milk education, acceptance, storage, and feeding are conducted in accordance with the evidence-based guidelines for breast milk feeding of hospitalized neonates in China [12].

EN: ① Initiation time: preterm infants without intrauterine distress and relatively stable conditions start feeding within 12 h after birth. Those with intrauterine distress or unstable conditions start feeding 12–24 h after birth, or it may be appropriately extended to 24–48 h. ② Initial feeding volume: very low birth weight infants (VLBWI) start with 1–2 ml, every 2 h; extremely low birth weight infants (ELBWI) start with 0.5–1 ml, every 2 h. ③ Milk volume increase plan: micro-feeding is carried out on days 1–4 after birth. If tolerated, the rate of milk increase for VLBW preterm infants is 20–30mL / (kg·d), and for ELBW preterm infants, it’s 15–25 ml / (kg·d), until the milk volume reaches 150–180 ml / (kg·d) or the calorie intake reaches 110–130 kcal / (kg·d) (1 kcal = 4.184 kJ).

Parenteral Nutrition (PN): Start PN support within 24 h after birth. Implement PN based on gestational age, weight, day of life, and clinical conditions. Adjust the PN dosage according to the volume of enteral feeding. Discontinue PN when the milk volume reaches 120mL/ (kg·d) or the calorie intake from EN reaches 90 kcal / (kg·d). Use the “all-in-one” parenteral nutrition solution uniformly configured by the hospital’s intravenous configuration center.

Start nasogastric tube feeding after birth and gradually transition to oral feeding at a corrected gestational age of 32–34 weeks.

### General information questionnaire

Databases such as CNKI, Wanfang database, VIP database and PubMed were searched, literatures related to breast milk feeding were read, variables were screened in combination with clinical practice and expert opinions, and a general data questionnaire was formulated, including the following two parts. ① Demographic data: infant sex, gestational age, birth weight, mode of delivery, Score for Neonatal Acute Physiology Perinatal Extension II (SNAPPE-II) [13], length of hospital stay, maternal education level, and paternal education level of the preterm infants. ② Growth and development assessment: length, weight, and head circumference at a corrected gestational age of 37 weeks.

### Test of infant motor performance(TIMP)

TIMP was established by American scholar Girolamil et al. [14] in 1983, and it is suitable for premature infants with a corrected gestational age of 34 weeks to infants with a corrected age of 4 months. The purpose is to evaluate the motor control, postural coordination and functional activity-related motor ability of premature infants and young infants, which has a predictive effect on the later motor development of infants. There are 42 items in the TIMP assessment, of which items 1 to 13 are observation items, including selective postural control, midline alignment, and movement quality. Items 14–42 were elicited items: including sitting position (items 14–18), supine position (items 19––27), turning over (items 28–31), lateral position (items 32–34), prone position (items 35–39), standing position (items 40–42) in terms of infant head, response to visual and auditory stimuli, defensive movements, trunk movement, limb movement, etc. In the first 13 observation items, if the baby achieves the corresponding performance, they will score 1 point; if there is no corresponding performance, the score will be 0 points. The last 29 induced items are graded from 0 to 6 points. The original score is obtained by adding the scores of 42 items (13 observation items + 29 eliciting items). The original score ranges from 0 to 142 points and takes 20 to 40 min. EPIs were assessed at 37 weeks corrected gestational age.

### Neonatal neurobehavioral assessment 

Neonatal neurobehavioral assessment (NBNA), a method for assessing the neurobehavior of newborns in China, was developed by Professor Bao Xiulan, an early childhood education expert and a professional at Beijing Union Medical College Hospital. This method integrates the strengths of the NBAS, theNeonatal Behavioral Assessment Scale, by Professor Brazelton from Boston University, USA, and the neonatal neuromotor assessment method by Amiel-Tison from France, combined with her own experience. The NBNA comprises 20 items and provides a comprehensive reflection of the functional state of the brain in newborns [15]. The NBNA comprises five parts: ① Behavioral ability (six items, including response to sound and light stimuli, startle response, facial response, response to red ball, comfort response); ② Passive muscle tension ( four items, including scarf sign, forearm rebound, popliteal angle, lower limb rebound ); ③ Active muscle tension ( four items, including neck flexor and extensor active contraction, hand grip, traction response and support response upright); ④ Primitive reflexes (three items, including sucking action, hugging reflex, step or place response); ⑤ General assessment (three items, including wakefulness, crying, and activity). The score for each item has three partitions: 0 points, 1 point and 2 points. The total score is 40 points, and the abnormal NBNA is defined as the total score ≤ 35 points. EPIs were assessed at 37 weeks corrected gestational age.

### Bedside cranial ultrasound examination

Using the portable color Doppler ultrasound diagnostic device, the M-turbo, manufactured by Sonosite with a probe frequency range of 4 to 8 MHz, specialized neonatal neurosonologists conduct regular bedside cranial examinations on EPIs during the first, second, third, and fourth weeks after birth, aiming to compile the cumulative incidence within the first four weeks.

### Data quality control

EPIs were evaluated by professional nurses with TIMP and NBNA operating qualifications in the neonatology department. Data receipts were collected by two systematically trained graduate students from January to March 2023 in strict accordance with the inclusion and exclusion criteria. General information on premature infants was collected by reviewing the electronic medical record system. The electronic medical record system was reviewed to ascertain the proportion of breast milk feeding; data were entered into ab Excel form after being checked by two people. Guidance from statistical experts was sought for any problems found during data analysis to ensure the accuracy of the data. A complete set of data were kept by a designated research team member.

### Statistical analyses

Statistical analysis was performed using SPSS 27.0 software. The measurement data conforming to the normal distribution is described by mean ± standard deviation, and the comparison between groups is carried out by one-way analysis of variance. Enumeration data were expressed as cases and percentages, and *χ2* test was used for comparison between groups. For dimensions with statistical significance, further post-hoc tests were performed, and *p* < 0.05 was considered statistically significant.

## Results

### Characteristics of sample

This study initially included 201 EPIs, with 9 deaths and 5 cases of incomplete data. Finally, 187 EPIs were included, consisting of 100 males (53.5%) and 87 females (46.5%). The gestational age ranged from 22.29 to 27.86 weeks (average 26.30 ± 1.29 weeks), and the birth weight varied from 450 to 1370 g (average 874.03 ± 195.24 g). The duration of hospital stay was between 49 and 169 days (average 76.44 ± 20.53 days). There were 71 cases (38%) of cesarean section and 116 cases (62%) of vaginal delivery. A low proportion of breast milk feeding was observed in 70 cases (37.4%), medium proportion breast milk feeding in 61 cases (32.6%), and high proportion breast milk feeding in 56 cases (29.9%).

#### Comparison of the basic conditions of the three groups of EPIs

The comparison of infant sex, gestational age, birth weight, mode of delivery, SNAPPE II, length of hospital stay, maternal education level, and paternal education level among the three groups of EPIs showed no statistically significant differences (*p* > 0.05). However, the differences in length and weight at a corrected gestational age of 37 weeks were statistically significant (*p* < 0.05), as shown in Table 1.

**Table 1:** Summary of the demographic characteristics of participants

Characteristic	Breast milk group			test statistic	*P-*value
	Low proportion (*n* = 70)	Medium proportion (*n* = 61)	High proportion group(*n* = 56)		
Infant sex [case (percentage, %)]				1.923^1^	0.382
males	33(47.1)	34(55.7)	33(58.9)		
female	37(52.9)	27(44.3)	23(41.1)		
Gestational age (weeks, ‾X ± s)	26.38 ± 1.25	26.37 ± 1.21	26.13 ± 1.43	0.726^2^	0.485
birth weight(g, ‾X ± s)	873.29 ± 195.05	912.46 ± 167.38	833.11 ± 217.42	2.450^2^	0.089
Mode of delivery [ case (percentage, %)]				1.565^1^	0.457
Cesarean section	25(35.7)	27(44.3)	19(33.9)		
vaginal delivery	45(64.3)	34(55.7)	37(66.1)		
SNAPPEII	22.94 ± 13.14	19.25 ± 11.48	24.16 ± 12.75	2.509^2^	0.084
Hospitalization days (days)	75.8 ± 19.67	73.59 ± 16.14	80.34 ± 25.11	1.644^2^	0.196
Maternal education level [case (percentage, %)]				4.681^1^	0.322
Primary and Junior High	15(21.4)	20(32.8)	11(19.6)		
High school, technical secondary school	24(43.3)	13(21.3)	18(32.1)		
Bachelor degree, college degree and above	31(44.3)	28(45.9)	27(48.2)		
Father ‘s education level [case (percentage, %)]				8.609^1^	0.072
Primary and junior high	19(27.1)	24(39.3)	9(16.1)		
High school, technical secondary school	25(35.7)	15(24.6)	20(35.7)		
Bachelor degree, college degree and above	26(37.1)	22(36.1)	27(48.2)		
Corrected head circumference at gestational age of 37 weeks (cm, ‾X ± s)	31.86 ± 1.55	31.33 ± 1.48	31.89 ± 1.69	2.476^2^	0.087
Corrected length at a gestational age of 37 weeks(cm, ‾X ± s)	45.94 ± 1.65	46.65 ± 1.39	47.13 ± 1.03	11.542^2^	<0.001
Corrected weight at a gestational age of 37 weeks(g, ‾X ± s)	2534.79 ± 132.21	2694.26 ± 254.44	2710.09 ± 218.66	14.652^2^	<0.001
Corrected head circumference Z-score at a gestational age of 37 weeks.	-0.96 ± 1.2	-1.36 ± 1.14	-0.93 ± 1.3	2.476^2^	0.087
Corrected length Z-score at a gestational age of 37 weeks	-1.29 ± 0.66	-0.93 ± 0.59	-0.72 ± 0.54	14.175^2^	<0.001
Corrected weight Z-score at a gestational age of 37 weeks	-1.05 ± 0.36	-0.62 ± 0.69	-0.58 ± 0.59	14.652^2^	<0.001

1χ2 value;2 F value

### TIMP comparison of three groups of EPIs

**Fig. 1:**
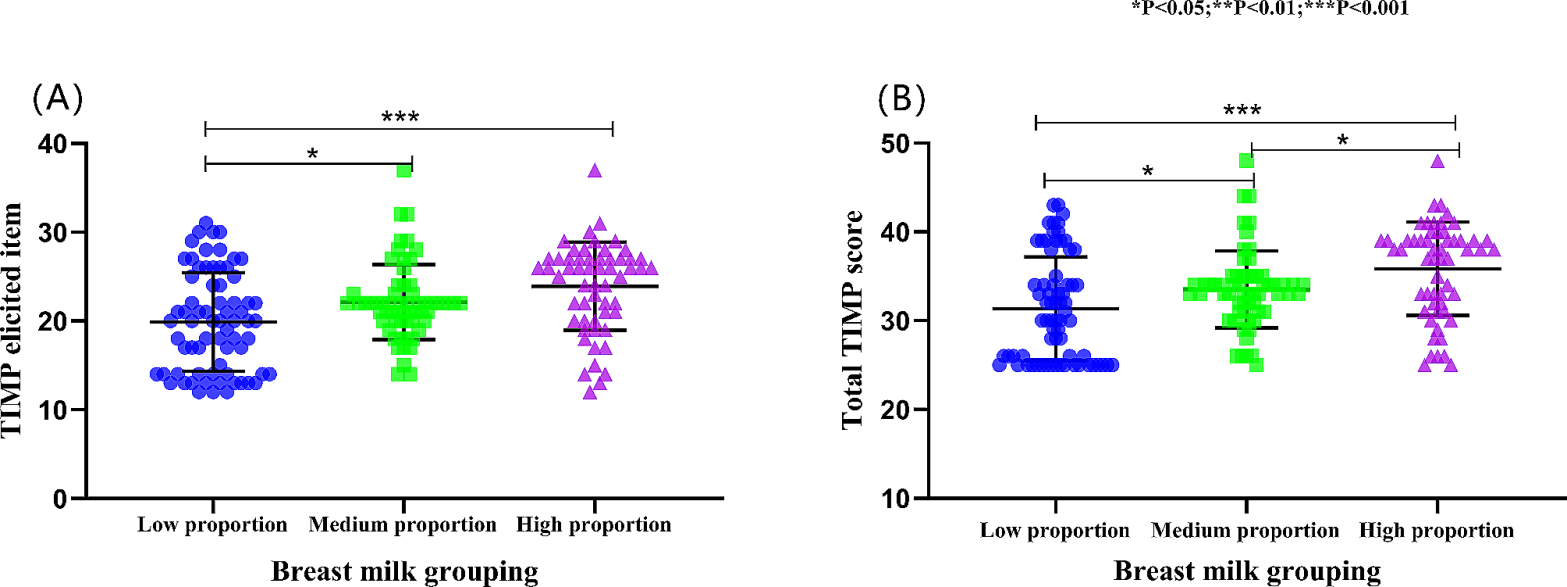
Chart of the relationship between breast milk volume and infant motor. TIMP: Test of infant motor performance

The TIMP elicited dimension and the total TIMP score of breast milk feeding in different proportions were statistically significant (*p* < 0.05), and there was no statistical difference between the observed items (*p* > 0.05) (see Table 2). For further post hoc comparison of TIMP elicited dimensions and total TIMP scores, the low proportion breast milk feeding group scored lower than the medium proportion breast milk feeding group and the high proportion breast milk feeding group for the elicited item dimensions, with statistically significant differences (*p* < 0.05), as shown in Fig. 1 (A). Regarding the total TIMP score, the total TIMP score of the low proportional breast milk feeding group was lower than that of the medium proportional breast milk feeding and high proportional breast milk feeding groups, and the total score of the medium proportional breast milk feeding group was lower than that of the high proportional breast milk feeding group, and the difference was statistically significant (*p* < 0.05), as shown in Fig. 1 (B).

**Table 2:** Comparison of infant motor performance outcomes at corrected gestational age of 37 weeks

Variable	Breast milk group			F value	*P-*value
	Low proportion (*n* = 70)	Medium proportion (n = 61)	High proportion (n = 56)		
Observation items	12 ± 0.76	11.97 ± 0.71	12.16 ± 0.76	1.133	0.324
Elicited items	19.89 ± 5.55	22.13 ± 4.22	23.91 ± 4.98	10.354	< 0.001
Total TIMP score	31.34 ± 5.85	33.52 ± 4.33	35.86 ± 5.27	11.661	< 0.001

*TIMP* test of infant motor performance

### NBNA comparison of three groups of EPIs

**Fig. 2:**
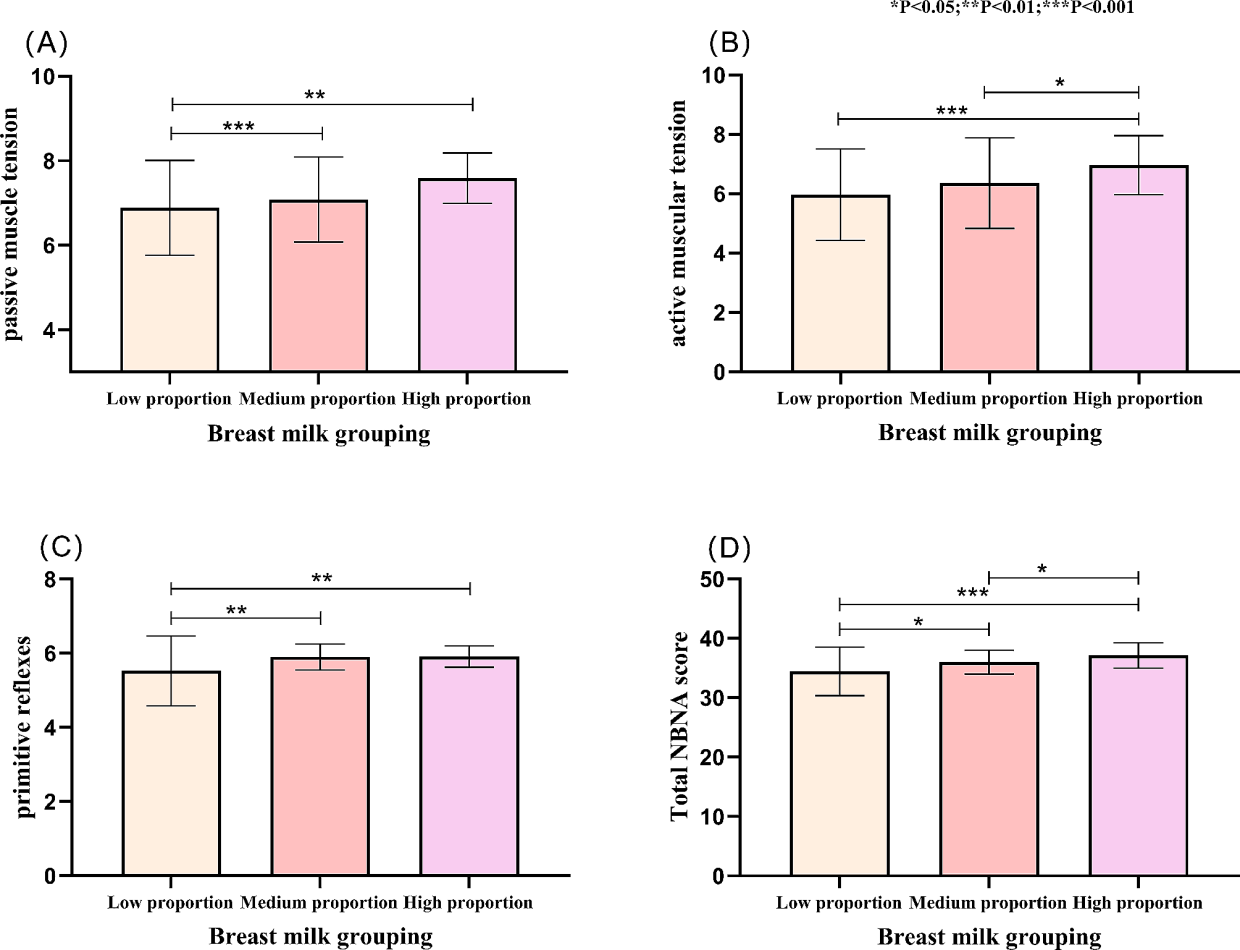
Chart of the relationship between breast milk intake and neonatal neurobehavioral outcomes. *NBNA* Neonatal neurobehavioral assessment

Passive muscle tension, active muscle tension, primitive reflexes and total NBNA scores were statistically significant (*p* < 0.05) in the NBNA of different proportions of breast milk feeding groups, and there was no statistical difference in behavioral ability and general assessment (*p* > 0.05) see Table 3. In terms of passive muscle tension and primitive reflexes, the scores of the low proportion breast milk feeding group were lower than those of the medium proportion breast milk feeding and high proportion breast milk feeding groups, with statistically significant differences (*p* < 0.05) as shown in Fig. 2 (A, C). In terms of active muscle tension, the scores of the high proportion breast milk feeding group were higher than those of the low and medium proportion breast milk feeding groups, with statistically significant differences (*p* < 0.05) as shown in Fig. 2 (B). For the total NBNA score, the low proportion breast milk feeding group had lower scores than the medium proportion breast milk feeding and high proportion breast milk feeding groups, and the difference was statistically significant (*p* < 0.05); The scores of the medium proportional breast milk feeding group were lower than those of the high proportional breast milk feeding group and the difference was statistically significant (*p* < 0.05) (see Fig. 2 (D)).

**Table 3:** Comparison of neonatal neurobehavioral assessment outcomes at a corrected gestational age of 37 weeks

Variable	Breast milk group			F value	*P* value
	Low proportion (*n* = 70)	Medium proportion (*n* = 61)	High proportion (*n* = 56)		
Behavioral ability	10.13 ± 1.78	10.64 ± 1.34	10.66 ± 1.63	2.318	0.101
Passive muscle tension	6.89 ± 1.12	7.08 ± 1.01	7.59 ± 0.6	8.779	<0.001
Active muscle tension	5.97 ± 1.54	6.36 ± 1.53	6.96 ± 0.99	7.911	<0.001
Primitive reflexes	5.53 ± 0.94	5.9 ± 0.35	5.91 ± 0.29	7.822	<0.001
General assessment	5.96 ± 0.27	5.98 ± 0.13	6 ± 0	0.933	0.395
Total NBNA score	34.43 ± 4.09	35.98 ± 2	37.13 ± 2.13	12.98	<0.001

*NBNA* Neonatal neurobehavioral assessment

### Comparison of cranial ultrasound results of three groups of EPIs

**Fig. 3:**
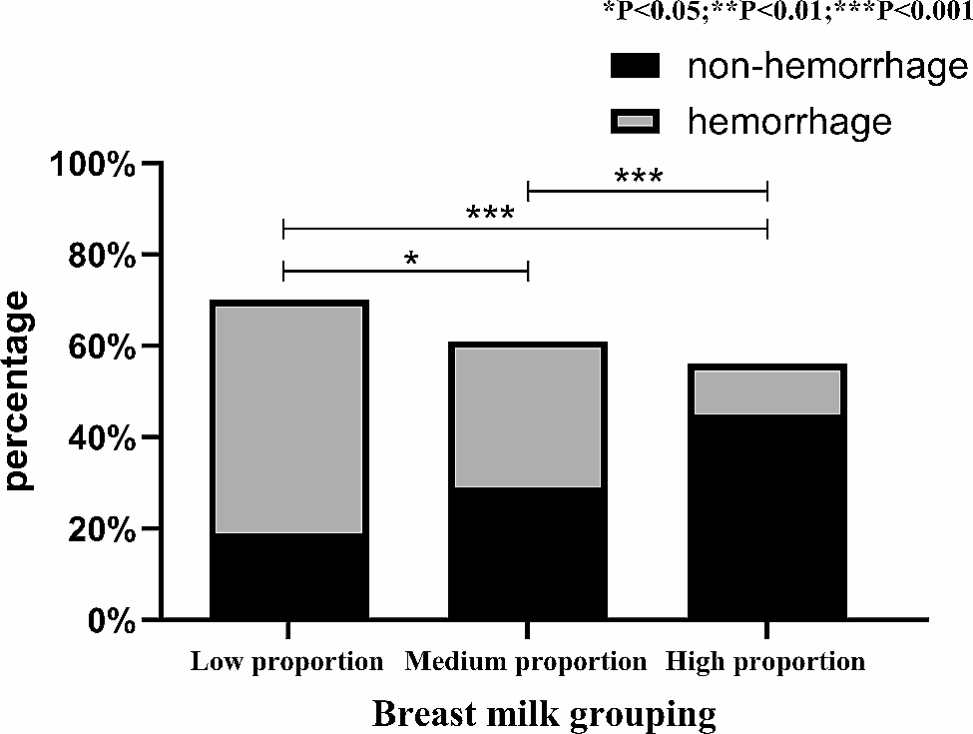
The relationship between breast milk intake and the incidence of intracranial hemorrhage detected by head ultrasound

Intracranial hemorrhage was statistically significant (*p* < 0.05) in the different proportional breast milk feeding groups as revealed by cranial ultrasound, and the hemorrhage rate in the high proportional breast milk feeding group was notably lower at 19.6% compared to the medium proportional breast milk feeding group at 52.5%, and the low proportional breast milk feeding group at 72.9%. Furthermore, the hemorrhage rate in the medium proportional breast milk feeding group was significantly lower than that in the low proportional breast milk feeding group (*p* < 0.05), as illustrated in Fig. 3.

## Discussion

Our findings resonate with those of a recent double-blind randomized controlled study, which demonstrated that fortifying breast milk with an optimal proportion of protein, carbohydrates, and fats significantly improved length, head circumference, fat, and lean mass in preterm infants at 36 weeks [16]. Adequate early postnatal protein and energy supply are crucial for brain growth and myelination [17]. A growth rate of 21.2 g / kg / d in preterm infants (from the time birth weight is regained to 2000 g) is associated with optimal neurodevelopmental outcomes [18]. This aligns with our findings, where infants fed with a medium-to-high proportion of breast milk exhibited better weight and length than those in the low proportion group, and their neurobehavioral development was also superior. This suggests that growth in weight and length can promote neurological development. However, in our study, the medium and high-proportion groups exhibited no differences in weight and length, yet neurobehavioral development showed the high proportion group was superior to the medium proportion group. This indicates that the promotion of neurological development by breast milk feeding is not solely achieved by providing adequate protein and energy.

The results of this study show that the elicited dimension scores of the low breast milk feeding group were lower than those of the medium and high breast milk feeding groups, indicating that medium and high levels of breast milk feeding led to better development of EPIs’ responses to visual and auditory stimuli, defensive actions, trunk movement, and limb movement. A study by Zhang et al., showed that compared with infants fed with formula milk, those fed with breast milk had a larger volume of gray matter, especially in the bilateral frontal lobes, left caudate nucleus, and right temporal lobe [19]. The frontal lobe plays an important role in many processes, such as motor control, urination, cognition, attention, memory, language, and neuropsychiatric functions, while the temporal lobe is related to auditory, visual, memory, and language functions [20]. A study by Khedr et al. demonstrated that at one year of age, breastfed infants exhibited more mature visual evoked potentials, auditory brainstem evoked potentials, and somatosensory evoked potentials compared to formula-fed infants. This suggests that breast milk feeding contributes to the early development and maturation of certain aspects of the nervous system when compared to infant formula milk [21]. The short-term impact of breast milk feeding on auditory perception needs further investigation. Belfort et al. evaluated the effects of breastfeeding on 180 preterm infants with gestational ages less than 30 weeks or birth weights less than 1250 g within 0─28 days after birth. The study found that the longer the number of days when breast milk volume exceeded 50% of EN, the larger the volume of deep gray matter at term-equivalent age [22]. An increase in regional gray matter may have a positive impact on future cognitive, behavioral, memory, and language development, but further research is needed to confirm this.

This study also showed that the passive muscle tension and primitive reflexes in the low-proportion breast milk feeding group were lower than those in the medium and high-proportion groups, further confirming the dose-response relationship between breast milk feeding and the development of the behavioral nervous system, which is positively correlated with feeding volume and duration [23]. This is consistent with the findings of Pineda et al. which showed a relationship between breast milk feeding and better behavioral neural outcomes [24]. Studies have shown that the neurotrophic factors in breast milk, including long-chain polyunsaturated fatty acids, cholesterol, sialic acid, taurine, hormones, and growth factors, as well as neuroprotective factors, such as glutamate, probiotics, and oligosaccharides, play important roles in the development of the nervous system and cannot be replaced by other milk products [25]. Long-chain polyunsaturated fatty acids and oligosaccharides in breast milk can promote brain development and myelination, improve white matter microstructure, increase cortical thickness through inflammation reduction, and promote neurite formation [8, 26]. The active muscle tone in the high-proportion breast milk feeding group was higher than that in the low and medium-proportion groups. Research has shown that the more breastfeeding, the stronger the ability to perform large muscle movements and solve problems, which is consistent with our findings [27]. However, our results show no differences in selective posture control, midline alignment, motor quality, or behavioral ability among high-proportion breast milk feeding groups, which needs further investigation. At the same time, only the total scores were different between the medium and high-proportion groups, with no differences in dimensions, which may be related to the small sample size of this study. It is suggested that more research is needed using a larger sample size.

The results of this study showed that the rate of cranial hemorrhage was lower in the high proportion breast milk feeding group than in the medium proportion breast milk feeding and low proportion breast milk feeding groups, and the rate of cranial hemorrhage was lower in the medium proportion breast mik feeding than in the low proportion breast milk feeding group. This is similar to the findings of Carome et al. [28]. This may be related to the important immune and anti-inflammatory roles of cytokines in breast milk, which can cross the intestinal barrier. Transforming growth factor β (TGF-β) is the most abundant cytokine family in breast milk, with higher levels in colostrum than in later preterm milk [29], It exerts a protective effect by lowering the levels of anticoagulant factors in the brain and stimulating the production of pericytes, providing a structural and maturing vascular system for the neurogenic matrix [30]. Additionally, epidermal growth factor (EGF) in breast milk, binds to EGF receptors (EGFR) in the brain and plays a role in astrocyte differentiation [31]. These cells provide strength and stability for the blood-brain barrier [32]. Zheng et al. performed a retrospective analysis on 604 very preterm infants (VPIs), discovering that a high proportion of breast milk feeding and breast milk feeding during the first week, accounting for over 50% of total intake, could reduce the likelihood of intraventricular hemorrhage (IVH) [33]. Torres et al. found that preterm infants who achieved full EN through exclusive breastfeeding within 7 days after birth had an 85% lower incidence of IVH compared to those who reached the same goal within 8 days [34]. However, the current rate of exclusive breast milk feeding among hospitalized preterm infants in China is not promising. Strengthening education for families during hospitalization and promoting breast milk feeding is recommended.

## Conclusions

Medium-to-high proportions of breast milk feeding can promote the behavioral neurodevelopment of EPIs, particularly in the early postnatal period. Further efforts to strengthen breast milk feeding management, enhance the establishment of breast milk banks, encourage NICUs to adopt breast milk feeding, increase the breast milk feeding rate, and promote the behavioral neurodevelopment of EPIs are encouraged.

### Limitations

This study is limited to a single-center investigation in a tertiary hospital within one city. Future multi-center, large-sample studies are recommended to further analyze the benefits of breast milk feeding. The proportion group (20–80%) in the breast milk feeding group has a large range. Lack of multivariate analysis for neurological impairment and IVH confounders further highlights the need for caution in interpretation. Subsequent research with refined methodology is encouraged to validate and extend these findings.

## Data Availability

The dataset is available from the corresponding author upon reasonable request.

## Abbreviations

EGF: Epidermal growth factor
EGFR: EGF receptors
ELBWI: Extremely low birth weight infants
EN: Enteral nutrition
EPIs: Extreme preterm infants
IVH: Intraventricular hemorrhage
NBNA: Neonatal behavior neurological assessment
NICU: Neonatal intensive care unit
PN: Parenteral nutrition
SNAPPE-II: Score for Neonatal Acute Physiology Perinatal Extension II
TGF-β: Transforming growth factor β
TIMP: Test of infant motor performance
VLBWI: Very low birth weight infants
VPIs: Very preterm infants
